# Agonist-induced calcium entry correlates with STIM1 translocation

**DOI:** 10.1002/jcp.20993

**Published:** 2007-06

**Authors:** Kehinde Ross, Michael Whitaker, Nick J Reynolds

**Affiliations:** Dermatological Sciences, Institute of Cellular Medicine, Medical School, Newcastle UniversityNewcastle upon Tyne, United Kingdom; Institute of Cell and Molecular Biosciences, Medical School, Newcastle UniversityNewcastle upon Tyne, United Kingdom

## Abstract

The mechanisms of agonist-induced calcium entry (ACE) following depletion of intracellular calcium stores have not been fully established. We report here that calcium-independent phospholipase A (iPLA_2_) is required for robust Ca^2+^ entry in HaCaT keratinocytes following ATP or UTP stimulation. Lysophosphatidic acid (LPA), an unrelated agonist, evoked Ca^2+^ release without inducing robust Ca^2+^ entry. Both LPA and UTP induced the redistribution of STIM1 into puncta which localized to regions near or at the plasma membrane, as well as within the cytoplasm. Plasma membrane-associated STIM1 remained high for up to 10 min after UTP stimulation, whereas it had returned almost to baseline by that time point in LPA-stimulated cells. This correlated with faster reloading of the endoplasmic reticulum Ca^2+^ stores in LPA treated cells. Thus by differentially regulating store-refilling after agonist-mediated depletion, LPA and UTP may exert distinct effects on the duration of STIM1 localization at the plasma membrane, and thus, on the magnitude and duration of ACE.

Tight control of free cytosolic calcium ([Ca^2+^]_i_) enables this second messenger to regulate diverse cell processes (Berridge et al., 2000). Receptor-mediated activation of phospholipase C (PLC) stimulates hydrolysis of phosphatidylinositol 4,5-bisphosphate (PIP_2_) into diacylglycerol (DAG) and inositol 1,4,5-trisphosphate (IP_3_) (Berridge et al., 2003). The latter evokes Ca^2+^ release via IP_3_ receptors (IP_3_Rs) on the endoplasmic reticulum (ER). This is usually followed by Ca^2+^ influx across the plasma membrane (PM), or agonist-induced Ca^2+^ entry (ACE) (Patterson et al., 2002). Similarly, depletion of ER stores by inhibition of the sarcoplasmic/endoplasmic reticulum Ca^2+^-ATPase (SERCA) with thapsigargin (TG) evokes store-operated Ca^2+^ entry (SOCE) (Parekh and Putney, 2005).

A mechanism for SOCE proposed by Bolotina and colleagues (Smani et al., 2004) involves a 600-Da diffusible factor “calcium influx factor” (CIF) of unknown identity that is released from the ER following store depletion (Randriamampita and Tsien, 1993; Bolotina and Csutora, 2005). CIF in turn activates calcium-independent phospholipase A (iPLA_2_) which generates lysophospholipids that activate SOCE at the PM by an uncharacterized process. In their studies, SOCE was impaired by knockdown of iPLA_2_ using RNA interference or by pharmacological inhibition of iPLA_2_ with bromoenol lactone (BEL), a specific iPLA_2_ inhibitor (Smani et al., 2004).

An alternative model for SOCE has emerged very recently, involving the re-organization of the Ca^2+^ sensor STIM1 into puncta which then activate Orai1/CRACM1, a transmembrane protein that appears to be the long-sought after SOCE channel (Feske et al., 2006; Prakriya et al., 2006; Vig et al., 2006; Yeromin et al., 2006). The STIM1–Orai1 complexes form in a spatially-restricted manner at ER-PM junctions and SOCE appears to occur predominantly in the vicinity of this nexus (Luik et al., 2006). In several independent studies, co-expression of Orai1 and STIM1 resulted in large (10–60-fold) increases in SOC currents (Mercer et al., 2006; Peinelt et al., 2006; Soboloff et al., 2006; Zhang et al., 2006). STIM1 itself is a 90-kDa phosphorylated transmembrane protein with an unpaired Ca^2+^-binding EF hand and sterile α motif (SAM) in the N-terminal domain (Manji et al., 2000; Williams et al., 2002). It localizes to the ER with its N-terminus buried in the lumen. Discharging the stores causes Ca^2+^ to dissociate from the N-terminal EF-hand, freeing STIM1 to translocate to the PM (or to puncta near the PM) (Liou et al., 2005; Roos et al., 2005; Zhang et al., 2005). Although cell surface STIM1 complexes were detected by surface biotinylation (Zhang et al., 2005), and pre-incubation with a monoclonal antibody directed against the extracellular domain of STIM1 blocked the Ca^2+^ release-activated Ca^2+^ current (*I*_CRAC_) in Jurkat T cells (Spassova et al., 2006), the extent to which STIM1 actually crosses the PM after store depletion is not clear (Mercer et al., 2006). Indeed in a very recent study using electron microscopy, Lewis and co-workers were unable to detect insertion of STIM1 into the PM (Wu et al., 2006). Notably, STIM1 also appears to associate with and activate TRPC1, a member of transient receptor potential family of cation entry channels (Huang et al., 2006; Lopez et al., 2006).

In this study, we have examined the role of iPLA_2_ and STIM1 in ACE in HaCaT keratinocytes. We have found that inhibition of iPLA_2_ with BEL impaired UTP and ATP-induced Ca^2+^ entry. We have also observed that stimulation with physiological agonists triggered restructuring of STIM1 into puncta that were assembled at or near the PM. The duration of STIM1 localization to the PM appeared to be agonist dependent, with UTP promoting sustained targeting of STIM1 to the PM whereas lysophosphatidic acid (LPA) induced only transient association of STIM1 with the PM. Together, our findings suggest that divergent signaling pathways differentially regulate ACE by controlling the duration of STIM1 localization to the PM.

## Materials and Methods

### Reagents

Fluo-4-AM was obtained from Invitrogen (Paisley, UK), bromoenol lactone (BEL) from Sigma (Poole, Dorset, UK). All other reagents, including MCDB153 medium were from Sigma unless indicated otherwise. The dsRed-ER vector was from Clontech (Mountain View, CA).

### Cell culture and nucleofection

HaCaT keratinocytes, a kind gift from Dr. NE Fusenig (German Cancer Research Center, Heidelberg), were grown in DMEM supplemented with 10% fetal calf serum (FCS) and antibiotics. The YFP-STIM1 expression construct was a generous gift from Tobias Meyer (Stanford University, Stanford, CA). Cells were nucleofected (Amaxa Biosystems, Cologne, Germany) according to the manufacturer's instructions. Briefly, 10^6^ cells were resuspended in 100 µl of nucleofection solution with 5 µg of YFP-STIM1 plasmids, transferred to a cuvette and nucleofected on program U20. The cells were then resuspended in 500 µl of complete medium and seeded in Willco glass-bottomed microwell dishes (Intracel, Royston, UK). In some experiments, transfections were performed with Lipofectamine Plus (Invitrogen (Paisley, UK)) according to the manufacturer's protocol.

### Calcium imaging

HaCaT keratinocytes were seeded in Willco glass-bottomed microwell dishes (Intracel, Royston, UK) the day prior to experimentation. Cells were loaded with 3 µM of Fluo-4 acetoxymethyl (AM) ester for 45 min in supplemented MCBD153 medium (Todd and Reynolds, 1998) with 70 µM Ca^2+^ unless indicated otherwise. To minimize compartmentalization of the dye, 200 µM of the anion transport inhibitor sulphinpyrazone in dimethylsulphoxide (DMSO) was included in the medium during loading and de-esterification (Di Virgilio et al., 1988). After loading, cells were washed in phosphate-buffered saline (PBS) and incubated in medium for 45–60 min at 37°C to allow de-esterification of the dye. Vehicle, or BEL at 10–20 µM, was added to the medium at a final concentration of 10–20 µM for the last 30 min of de-esterification. Fluorescence quenching assays were performed by adding MnCl_2_ (prepared in PBS) at a final concentration of 500 µM. For Ca^2+^-free assays, cells were loaded as above using nominally Ca^2+^-free Krebs-Henseleit buffer.

Changes in [Ca^2+^]_i_ were monitored at 4-sec intervals with a Leica TCS SP2 confocal laser scanning microscope equipped with an argon laser (Leica, Milton Keynes, UK). A heated stage was used to maintain the cells at 37°C during image acquisition, and images were captured using a 63× Plan Apo objective (NA1.32). Fluo-4 was excited with the 488-nm line of the laser, collecting emitted fluorescence through a 500–550 nm window of the detector. Quantification was performed with Leica confocal software, and changes in [Ca^2+^]_i_ expressed as the ratio of the initial fluorescence to the temporal fluorescence (F_t_/F_0_).

### Analysis of STIM1 translocation

The cells were washed 2–3 times in nominally Ca^2+^-free KHB prior to visualization. Images of YFP-STIM were then acquired in nominally Ca^2+^-free KHB at 15-sec intervals using a 514-nm laser line for YFP excitation, and capturing YFP emission through a 525–600-nm window. Cells were stimulated with UTP or LPA about 25 sec after the start of recording. The fold increase in YFP fluorescence at the plasma membrane compared to the cytosol was estimated as follows: a 2 × 2 µm box was drawn in the cytosol while PM was determined by defining a small region of interest that had little or no visually discernable pre-formed complexes. The ratio of the PM/cytosolic pixel intensities was calculated and normalized to the pre-stimulation (t = 0) ratio.

### Statistical analysis

Results of the Ca^2+^ imaging experiments are presented as means (±SEM) which were determined in GraphPad Prism or Microsoft Excel. Statistical significance was determined using the unpaired two-tailed Student's *t*-test (GraphPad Prism or Microsoft Excel). Results with *P* < 0.05 were considered significant.

## Results

### UTP induces Ca^2+^ entry in keratinocytes

**Fig. 1:**
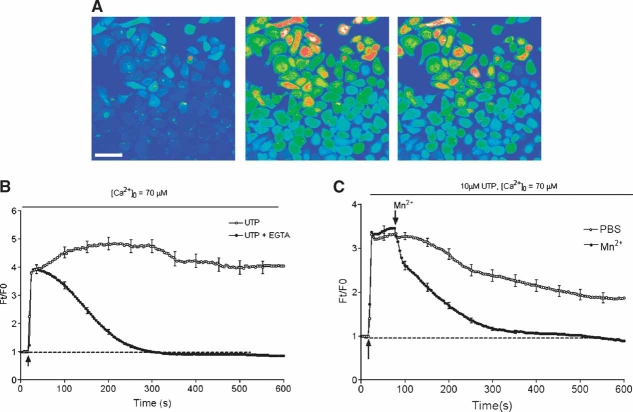
Agonist-induced Ca^2+^ entry (ACE) in HaCaT keratinocytes. HaCaT keratinocytes were loaded with Fluo-4 and imaged in medium with 70 µM [Ca^2+^]_o_ as described under “Methods.” **A**: Pseudocolor confocal micrographs showing [Ca^2+^]_i_ elevation in cells stimulated with 10 µM UTP. Images presented are before, 20 sec and 10 min after stimulation. Scale bar, 47 µm. **B**, **C**: The mean changes in [Ca^2+^]_i_ are presented as F_t_/F_0_ ratios (temporal fluorescence intensity/initial fluorescence intensity). B: Sustained [Ca^2+^]_i_ elevation was abolished by EGTA (*open circles*, 10 µM UTP, n = 31 cells; *filled circles*, 10 µM UTP plus 10 mM EGTA, n = 34 cells). C: Quenching of Fluo-4 fluorescence with Mn^2+^ (500 µM, *closed circles*, n = 39 cells) but not in control cells (n = 37 cells, *open circles*) to which PBS was added. For clarity, only selected error bars (SEM.) are shown. Data are representative of three independent experiments.

Extracellular nucleotides enhance proliferation of keratinocytes and other cells (Burrell et al., 2003). Stimulation of HaCaT keratinocytes with ATP or UTP induces [Ca^2+^]_i_ oscillations of decreasing amplitude in the absence of external Ca^2+^ (Burrell et al., 2003). With only 70 µM extracellular Ca^2+^ ([Ca^2+^]_o_) in the medium, we observed that stimulation with UTP evoked a rapid increase in [Ca^2+^]_i_ that remained elevated for the duration of recording (Fig. 1A), suggesting that UTP induced Ca^2+^ entry. To confirm that the sustained [Ca^2+^]_i_ elevation was due to Ca^2+^ entry (and not for instance inhibition of Ca^2+^ pumps), paired assays were performed in which cells were stimulated with UTP alone or UTP and the Ca^2+^ chelator EGTA. Simultaneous addition of UTP and EGTA resulted in a gradual return of the [Ca^2+^]_i_ to basal levels (Fig. 1B), indicating that chelation of [Ca^2+^]_o_ abolishes the sustained [Ca^2+^]_i_ plateau. Similar results were obtained with ATP (data not shown).

To provide further evidence that the agonist-induced elevation of [Ca^2+^]_i_ was due to Ca^2+^ entry across the PM, we performed fluorescence quenching assays in which Mn^2+^ was added to the medium after stimulation. Addition of Mn^2+^ led to a return of the [Ca^2+^]_i_ signal baseline levels (Fig. 1C). Taken together, these data confirm that stimulation of HaCaT keratinocytes with UTP results in Ca^2+^ entry across the plasma membrane.

### Inhibition of iPLA_2_ impairs ACE

**Fig. 2:**
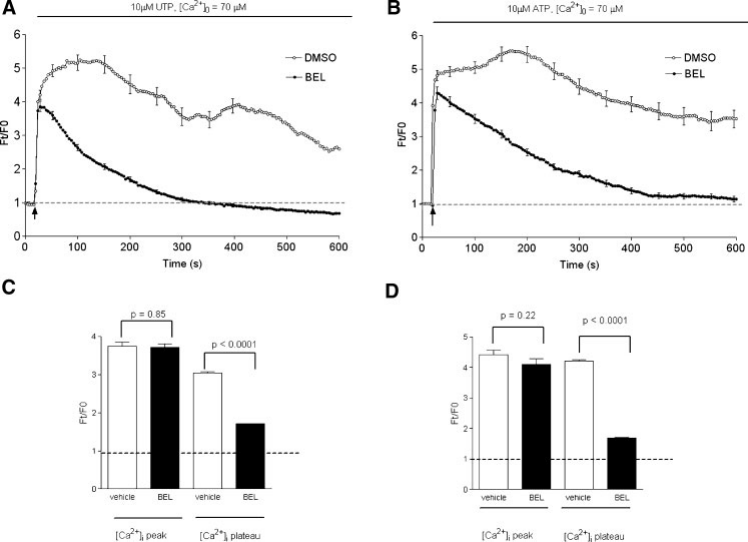
Pharmacological inhibition of iPLA_2_ impairs ACE. Averaged traces from paired assays showing the mean changes in [Ca^2+^]_i_ in HaCaT keratinocytes treated with the iPLA_2_ inhibitor BEL (*filled circles*), or with vehicle (*open circles*) for 30 min before imaging. The cells were stimulated with (**A**) 10 µM UTP, or (**B**) 10 µM ATP, as indicated. Numbers of cells (*n*) averaged: (A) BEL 35, DMSO 34; (B) BEL 25, DMSO 23. (**C**, **D**) Summary data pooled from three to four independent experiments (n = 104–135 cells). Plateau phases were averaged over 100 sec.

Inhibition of iPLA_2_ with BEL, a specific pharmacological inhibitor with a 1000-fold selectivity for iPLA_2_ over cytosolic (85 kDa) PLA_2_ (Hazen et al., 1991) has been shown to impair SOCE (Smani et al., 2004). However, electrophysiological measurements suggest that TG-induced SOCE might not be mediated by the same channels that mediate IP_3_-activated SOCE (Vanden Abeele et al., 2004). Therefore, we investigated whether iPLA_2_ activity was required for ACE. As shown in Figure 2A, treatment of cells with BEL led to a rapid decline in the [Ca^2+^]_i_ signal following stimulation with UTP, whereas [Ca^2+^]_i_ remained elevated in control cells. Similar results were obtained when the cells were stimulated with ATP (Fig. 2B). In addition to inhibiting iPLA_2_, BEL has also been reported to inhibit Mg^2+^-dependent phosphatidate phosphohydrolase (PAP-1), an enzyme involved in DAG turnover (Balsinde and Dennis, 1996). Treatment of cells with the PAP-1 inhibitor, propranolol (150 µM, 30 min preincubation (Fuentes et al., 2003)), did not impair ACE (data not shown). Thus the effects of BEL on ACE can be attributed to its inhibition of iPLA_2_ and not PAP-1.

### LPA induces Ca^2+^ release but not sustained Ca^2+^ entry

**Fig. 3:**
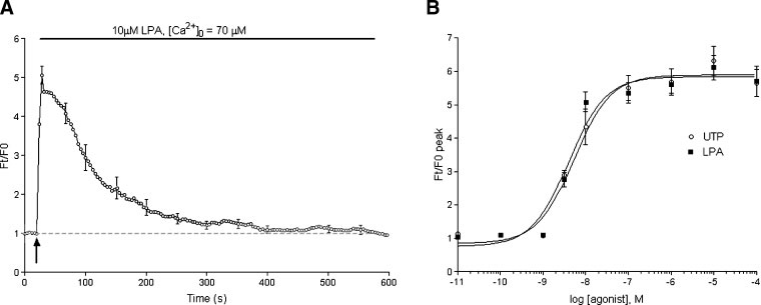
LPA-induces [Ca^2+^]_i_ release but not robust Ca^2+^ entry. **A**: HaCaT keratinocytes were stimulated with 10 µM LPA at the time point indicated by the *arrow*. Trace shown was averaged from n = 32 cells from one experiment, and similar results were obtained in four independent experiments. **B**: Dose-response curves for UTP (*open circles*) and LPA (*filled squares*). Results are the means ± SEM of the peak F_t_/F_0_ ratio determined from 26 to 46 cells.

LPA evokes Ca^2+^ release in many cells and modulates keratinocyte growth and migration (Mills and Moolenaar, 2003; Sauer et al., 2004). We stimulated cells with LPA to determine if it evoked Ca^2+^ entry. As shown in Figure 3, although Ca^2+^ release was observed, this was not followed by an elevated plateau, even though the extent of Ca^2+^ release (initial Ft/F0 peak) was similar to that obtained with UTP and ATP (compare Figs. 3A with 2A,B). Thus LPA does not appear to induce significant Ca^2+^influx in HaCaT keratinocytes. We have obtained similar results on primary normal epidermal keratinocytes.^1^ The same observations have been made on T cells and fibroblasts (Takemura et al., 1996; Waldron et al., 1997). The inability of LPA to stimulate robust Ca^2+^ entry compared to UTP was not due to differences in agonist potency, since a dose response assay showed that UTP and LPA were essentially equipotent (Fig. 3B), with EC_50_ values of 4.0 nM and 5.4 nM respectively.

### STIM1 translocation

**Fig. 4:**
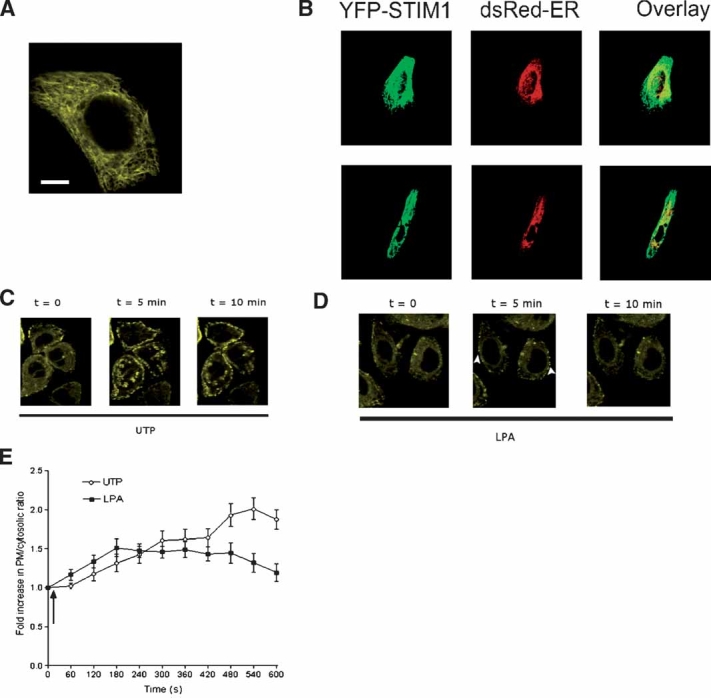
Differential kinetics of agonist-induced translocation of YFP-STIM1. **A**: Confocal micrograph depicting localization of YFP-STIM1 in a HaCaT keratinocyte. *Scale bar* = 10 µm. **B**: Colocalization of YFP-STIM1 and dsRed-ER. **C**, **D**: YFP-STIM1 redistribution in nominally Ca^2+^-free KHB was monitored in real time by confocal microscopy after stimulation with 10 µM UTP (C) or 10 µM LPA (D). Drugs were added 25 sec after the start of recording. **E**: Membrane accumulation of YFP-STIM1 was quantified as pixel intensity and normalized to the cytoplamsic YFP levels (see Methods for details). Data were pooled from three independent experiments (n = 18–21 cells).

Recently, STIM1 has been identified as a key mediator of SOCE (Liou et al., 2005; Zhang et al., 2005). When YFP-STIM1 was expressed in HaCaT keratinocytes, a reticular distribution was observed (Fig. 4A), similar to the pattern observed in other cell types (Liou et al., 2005). Co-expression of an ER marker (dsRed-ER) confirmed that YFP-STIM1 localized predominantly to the ER (Fig. 4B). However, YFP-STIM1 was also observed in regions where little or no ER staining was detected, notably at the extremities of the cells. Thus in resting cells, YFP-STIM1 appears to localize both to the ER and to other subcellular domains.

Next we tested the ability of UTP and LPA to promote STIM1 redistribution. These experiments were performed in Ca^2+^-free buffer to avoid any potential effects of Ca^2+^ entry itself on the spatiotemporal dynamics of STIM1. Both agonists induced the assembly of YFP-STIM1 puncta, which were formed within the cytoplasm and also at PM (Fig. 4C,D). This pattern is consistent with that observed by Meyer and colleagues on HeLa cells treated with histamine and thapsigargin (Liou et al., 2005). The lifetimes of the puncta were generally shorter upon LPA stimulation compared to UTP stimulation (compare Fig. 4C, D). Consistent with this, PM-proximal puncta levels continued to rise for almost 10 min after UTP stimulation (Fig. 4E). In contrast, YFP-STIM1 was not retained at the PM of LPA-treated cells. Instead, after reaching a peak about 3 min after stimulation, YFP-STIM1 appeared to return to the cytoplasm, such that by 10 min PM-associated YTP-STIM1 had returned to near-baseline levels. Thus the differential abilities of UTP and LPA to promote Ca^2+^ entry appear to be related to STIM1 translocation.

### Differential store refilling

**Fig. 5:**
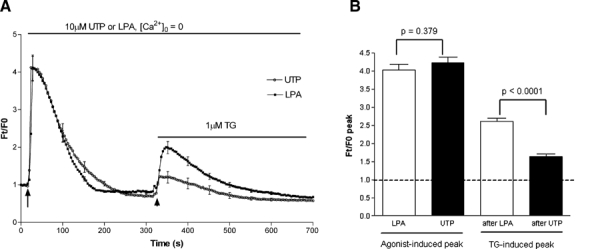
Differential store refilling after agonist-induced Ca^2+^ release. **A**: HaCaT keratinocytes loaded with Fluo-4 in nominally Ca^2+^-free KHB were stimulated with 10 µM of UTP (*open circles*, n = 27 cells) or LPA (*filled squares*, LPA n = 32 cells) as indicated by the *arrow*. After the Ca^2+^ signal had declined to baseline, cells were stimulated with 1 µM TG (*arrow head).* Data are from one representative experiment, similar results were obtained in three independent experiments. **B**: The peaks of the agonist-induced or TG-induced Ca^2+^ signals were pooled from three independent experiments (n = 112 cells in each case).

According to the current model, STIM1 is held predominantly in the ER with its unpaired Ca^2+^-binding EF hand in the ER lumen. Store depletion causes dissociation of Ca^2+^ from the EF hand, evoking STIM1 translocation. Our findings suggested that STIM1 starts migrating from the PM back to the ER shortly after LPA treatment, and this correlates with a reduction in Ca^2+^ entry (see Figs. 4D and 3A). Given that store refilling is thought to contribute to deactivation of Ca^2+^ influx and *I*_CRAC_ (Parekh and Putney, 2005), we speculated that Ca^2+^ stores may be reloaded more rapidly post-LPA stimulation compared to UTP stimulation. To test this hypothesis, cells were stimulated with agonists in a nominally Ca^2+^-free buffer. After the initial transient had returned to baseline, the cells were then treated with TG to empty the stores of residual Ca^2+^. As shown in Figure 5, the TG-induced Ca^2+^ peak after LPA treatment was significantly higher than that obtained after UTP. Thus by differentially regulating the re-filling of the Ca^2+^ stores, physiological agonists appear to control the duration of STIM1 localization to the PM and thus the magnitude and duration of ACE.

## Discussion

The activation of Ca^2+^ entry is the predominant mechanism for sustained elevation of [Ca^2+^]_i_ in non-excitable cells. The fundamental elements of SOCE are only now beginning to be defined. Studies by various groups have indicated that iPLA_2_ activity is required for SOCE (Smani et al., 2004; Vanden Abeele et al., 2004). In the present work, we have shown that iPLA_2_ activity is required for sustained ACE in HaCaT keratinocytes stimulated with extracellular nucleotides. Although experimental store depletion is often achieved by inhibition of the SERCA pump with TG, our observations indicate that iPLA_2_-mediated Ca^2+^ influx is likely to be functional in response to physiological agonists, not just TG. Thus even though Prevarskaya and colleagues found that the Ca^2+^ current generated by store depletion with TG was significantly more sensitive to BEL treatment than that generated by IP_3_ (Vanden Abeele et al., 2004), we argue in the present study that robust ACE in HaCaT keratinocytes is dependent on iPLA_2._ Both arachidonic acid and lysophospholipid products of iPLA_2_ activity have been implicated in Ca^2+^ entry (Smani et al., 2004; Mignen et al., 2005). However, arachidonic acid-mediated Ca^2+^ influx seems to function predominantly at low agonist concentrations (Shuttleworth et al., 2004). Therefore, given that our experiments were performed with supramaximal agonist concentrations of 10 µM ATP or UTP (see Fig. 3B), it is unlikely that Ca^2+^-selective arachidonate-regulated channels were significantly active.

Extracellular nucleotides signal through the P2Y family of G protein-coupled receptors (GPCR) (White and Burnstock, 2006). In addition, ATP also activates the P2X family of ion channels (White and Burnstock, 2006). However, given the similarity in the [Ca^2+^]_i_ dynamics of ATP and UTP-treated cells, the contribution of P2X channels to ATP-induced Ca^2+^ entry under our experimental conditions was arguably minimal.

In our investigations, we found that UTP and ATP evoked greater Ca^2+^ entry compared to LPA. This suggested that even though the respective UTP and LPA signaling pathways were equipotent for Ca^2+^ release on HaCaT keratinocytes (Fig. 3B), they were differentially coupled to Ca^2+^ entry. Given the recent identification of STIM1 as a Ca^2+^ sensor in the ER, we speculated that this might be related to divergent effects on the spatiotemporal dynamics of STIM1. Examination of YFP-STIM1 kinetics in the absence of [Ca^2+^]_o_ revealed striking puncta formation at the PM as well as in the cytoplasm. Exogenous UTP or LPA promoted translocation of YFP-STIM1 to the PM in the absence of [Ca^2+^]_o_, confirming that STIM1 translocation is likely to be a cause rather than consequence of ACE. The bulk of YFP-STIM1 persisted at the PM for up to 10 min following UTP stimulation (Fig. 4C,E) and in some experiments, up to 15 min. In contrast, the re-organization of YFP-STIM1 induced by LPA appeared to be relatively transient, and puncta did not persist at the PM for as long as those generated by UTP stimulation. This is the first evidence of differential regulation of agonist-induced STIM1 redistribution and suggests that PLC-activating agonists can be classified into those that promote sustained localization of STIM1 puncta to the PM, and those for which puncta formation is relatively short-lived. Interestingly, even though PM levels of STIM1 were similar for UTP and LPA at 5 min (Fig. 4E), [Ca^2+^]_i_ was significantly higher at that time point for UTP (compare Figs. 2A and 3A). This difference suggests that other pathways activated by LPA may exert negative feedback on Ca^2+^ entry at the STIM1-Orai1 nexus.

Several studies have shown that PLA_2_ activity is required for a range of intracellular trafficking events, such as retrograde membrane trafficking from the Golgi and *trans*-Golgi network (TGN) to the ER, and endocytic recycling of transferrin receptors (de Figueiredo et al., 2000, 2001). The redistribution of STIM1, however, appears to be independent of iPLA_2_ activity as incubation of cells expressing YFP-STIM1 with BEL for 30 min did not have any discernable effect on the translocation of YFP-STIM1 (data not shown). This does not, however, imply that BEL treatment has no effect on STIM1-mediated Ca^2+^ entry. For instance, BEL (that is, inhibition of iPLA_2_) could potentially inhibit the fundamental mechanism, as yet unknown, by which STIM1 activates Orai1. Further investigations will be required to establish whether there is any crosstalk between iPLA_2_ and STIM1-dependent Ca^2+^ entry.

Why do the Ca^2+^ stores seem to be reloaded more rapidly after LPA stimulation compared to UTP stimulation? Significant amounts of the Ca^2+^ released from intracellular reservoirs are extruded from cells by the plasma membrane Ca^2+^-ATPase (PMCA)(Parekh and Penner, 1997). Inhibition of PMCA would impair this extrusion, leaving more residual [Ca^2+^]_i_ for recharging of the stores by SERCA pumps. Thus differential regulation of PMCA activity may explain the observed differences in store refilling after LPA and UTP stimulation. This may be mediated by differential production of H_2_O_2_, an inhibitor of PMCA (Zaidi et al., 2003; Redondo et al., 2004). Both LPA and TG can induce H_2_O_2_ production in HaCaT cells (Sekharam et al., 2000). Alternatively, it is possible that UTP and LPA signaling pathways have distinct effects on tyrosine phosphorylation of PMCA, a modification that also impairs its activity (Dean et al., 1997). Determination of the relative contribution of PMCA activity to the clearance of [Ca^2+^]_i_ after agonist stimulation may help shed further light on the differential kinetics of UTP and LPA-induced Ca^2+^ signaling.

**Fig. 6:**
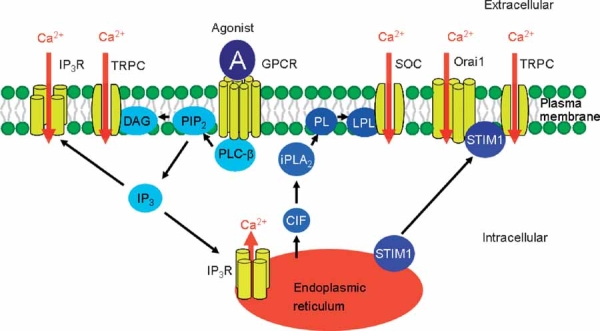
Schematic representation of an agonist-induced Ca^2+^ influx network. Activation of G protein-coupled receptors (GPCR) by an agonist (**A**) stimulates phospholipase C-β (PLC-β) activity via G proteins (not shown). The diacylglycerol (DAG) and inositol 1,4,5-trisphosphate (IP_3_) molecules subsequently generated by the hydrolysis of phosphatidylinositol 4,5-bisphosphate (PIP_2_) can activate Ca^2+^ entry through TRPC channels and cell surface IP_3_ receptors (IP_3_R), respectively. Discharge of endoplasmic reticulum Ca^2+^ stores by IP_3_ appears to trigger Ca^2+^ influx via at least three distinct pathways: CIF-iPLA_2_, STIM1-Orai1, and STIM1-TRPC. See text for details. For clarity, the Ca^2+/^calmodulin complex that CIF displaces from iPLA_2_ is not shown. CIF, Ca^2+^ influx factor; iPLA_2_, Ca^2+^-independent phospholipase A_2_; PL, phospholipids; LPL, lysophophosholipids; TRPC, canonical transient receptor potential channel.

The current understanding of ACE implicates a variety of cell surface receptors, phospholipases, ion channels, small molecules, and regulatory proteins in the activation, first of store depletion, and then of Ca^2+^ influx. We can consider these moieties as functional elements of a complex Ca^2+^ influx network (CaIN) that provides the cell with robustness with respect to Ca^2+^ entry. In this model, we envisage several routes to robustness, each designed to promote Ca^2+^ entry following PLC activation, regardless of variables in other parts of the CaIN. Thus, as illustrated in Figure 6, we can consider the ability of store depletion to (a) promote STIM1 translocation and coupling to Orai1 and TRPC proteins (Huang et al., 2006; Lopez et al., 2006), (b) trigger CIF-iPLA_2_ activation, and (c) evoke substantial Ca^2+^ entry through cell surface IP_3_Rs channels (Dellis et al., 2006) as well as the ability of DAG to activate Ca^2+^ entry through TRP channels (see Vazquez et al., 2004), altogether help confer highly optimized tolerance (Carlson and Doyle, 2002) on the CaIN.

In conclusion, we have shown in this study that iPLA_2_ activity is required for ACE, and that UTP-induced Ca^2+^ entry in HaCaT keratinocytes is associated with remodeling of STIM1 into puncta. The duration of STIM1 puncta localization to the PM appears to be agonist-dependent, with UTP but not LPA promoting sustained re-organization of STIM1. Further investigations will be required to clarify the basis for differential spatiotemporal dynamics of STIM1 in response to stimulation with Ca^2+^ mobilizing agonists.

## Footnotes

1 K.R, Graeme Parker, M.W, NJR; manuscript submitted.

## Abbreviations:

ACE: agonist-induced Ca^2+^ entry
BEL: bromoenol lactone
[Ca^2+^]_o_: extracellular Ca^2+^
[Ca^2+^]_i_: free cytosolic Ca^2+^
CIF: Ca^2+^ influx factor
DAG: diacylglycerol
ER: endoplasmic reticulum
*I*_CRAC_: Ca^2+^ release-activated Ca^2+^ current
iPLA_2_: Ca^2+^-independent phospholipase A_2_
KHB: Krebs-Henseleit buffer
PM: plasma membrane
SOCE: store-operated Ca^2+^ entry
TG: thapsigargin.
